# Infant in extremis: respiratory failure secondary to lower airway infantile hemangioma

**DOI:** 10.1186/s12887-022-03821-1

**Published:** 2022-12-30

**Authors:** Matthew S. MacDougall, Sarah Y. Afzal, Michael S. Freedman, Peggy Han

**Affiliations:** 1 Lucile Packard Children’s Hospital, Palo Alto, California, USA; 2 Stanford University School of Medicine, Department of Pediatrics, Palo Alto, California, USA; 3 Stanford University School of Medicine, Department of Pediatrics, Division of Pediatric Critical Care Medicine, Palo Alto, California, USA

**Keywords:** Vascular malformation, Bronchial compression, Dermatology, Otolaryngology, Case report

## Abstract

**Background:**

Infantile hemangiomas (IHs) are vascular tumors that commonly affect infants and usually regress spontaneously or can be easily treated as an outpatient with topical beta-blockers. However, IHs that present in the airway may cause life-threatening symptoms due to airway obstruction or risk of bleeding. Here we present the first documented case of an infant with rapid deterioration and acute respiratory failure secondary to a lower airway hemangioma.

**Case presentation:**

This 3-month-old male initially presented in respiratory distress with symptoms consistent with a viral respiratory infection, however showed no clinical improvement with standard therapies. An urgent CT scan revealed a mass occluding the right mainstem bronchus. Upon transfer to a tertiary care facility, he developed acute respiratory failure requiring emergent intubation and single lung ventilation. The availability of multiple subspecialists allowed for stabilization of a critically ill child, expedited diagnosis, and ultimately initiation of life-saving treatment with beta blockers. After 17 total hospital days, he was extubated successfully and discharged home in good condition.

**Conclusions:**

While IH is a rare cause of infantile respiratory distress, we present multiple pearls for the general pediatrician for management of IHs of the airway.

## Background

The biology and pathogenesis of infantile hemangiomas (IHs) are complex and not completely understood. IHs are vascular tumors with unclear origin characterized by early proliferation, followed by quiescence, then complete involution usually by the age of 7 [1].

IHs affect about 4-10% of infants [1], with the majority being solitary lesions (77%) and restricted to the head and neck (44%) [2]. Lower risk IHs are treated with observation and/or topical timolol therapy and do not require hospitalization [3, 4]. IHs can have high risk features, particularly in locations such as the face, eyes, and lips that may lead to disfigurement or functional impairment. Additionally, some IHs present with life threatening symptoms such airway obstruction, risk of bleeding (particularly those localized to the liver), or cardiac failure (when numerous). These high risk and life threatening IHs require early referral to IH specialists. Propranolol is the first line therapy for such lesions. The epidemiology and management of IHs are more extensively reviewed in clinical practice statements from pediatrics and dermatology [1, 4, 5].

With this case report, we present a unique encounter with a patient in severe respiratory distress from an obstructive lower airway infantile hemangioma. Our goal is to highlight clinical pearls to facilitate rapid recognition, initiation of treatment, involvement of multiple subspecialties, and expectations for safe de-escalation of care.

Airway hemangiomas, specifically those causing airway obstruction account for about 1.4% of IHs [3]. Airway IH most often affect the larynx or upper airway in the sub-glottic region [6, 7]. Upper airway IH often present with respiratory distress and biphasic stridor, and are often confused for croup or bronchiolitis [6]. Conversely, the incidence of lung and lower airway IHs is low and have mainly been described in limited case reports [8–11]. Furthermore, given their vascular character and rapid early proliferation phase, they frequently require escalation to critical care services, especially in the setting of hemorrhage and/or mass effect. Airway obstruction occurs by IH infiltration of the intra-luminal or extra-luminal bronchus, causing compression and hyperinflation of the affected side resulting in acute respiratory failure [8, 9, 11]. With this rare case, we hope to address key considerations in recognizing airway hemangioma as an important “can’t miss” differential diagnosis for the general pediatrician in a child with acute respiratory distress.

## Case presentation

A previously healthy 3-month-old male presented to a local community hospital in acute respiratory distress and was ultimately found to have an infantile hemangioma obstructing the lumen and causing extrinsic compression of the right mainstem bronchus. The patient presented to his pediatrician with a 4-day history of cough, congestion, increased work of breathing while sleeping and intermittent wheezing with feeds. He did not have any fevers, emesis, nor skin hemangiomas/rashes. He was initially diagnosed with a viral respiratory infection and sent home with supportive care. He re-presented to his general pediatrician with worsening respiratory distress and was referred to the emergency department. Upon arrival, he was noted to have grunting, nasal flaring, and significant retractions. He was immediately placed on 6 l per minute of high-flow nasal cannula with improvement in his respiratory distress and admitted to the pediatric ward for further management.

**Fig. 1:**
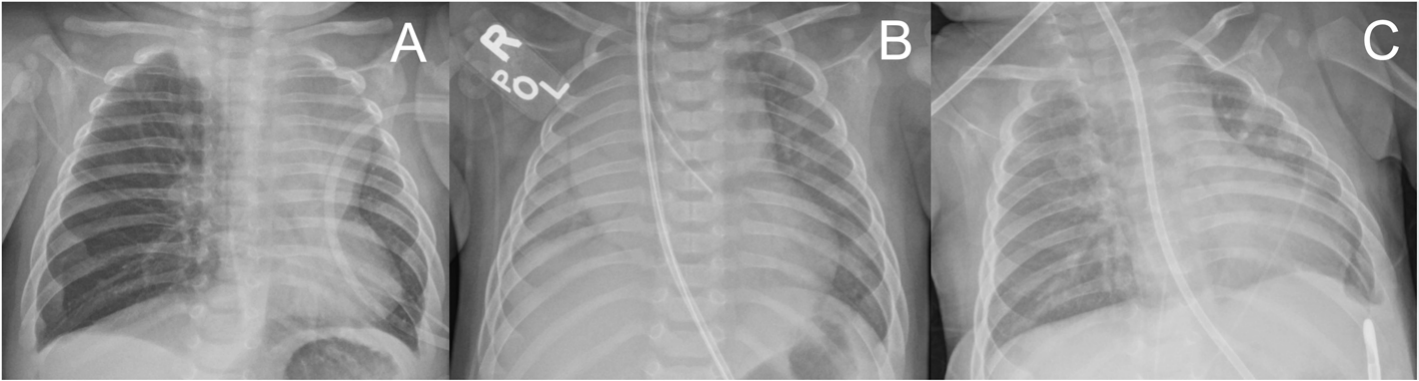
Serial chest x-rays from (**A**) initial presentation, (**B**) after left-mainstem intubation, and (**C**) after 10 days of treatment and extubation

**Fig. 2:**
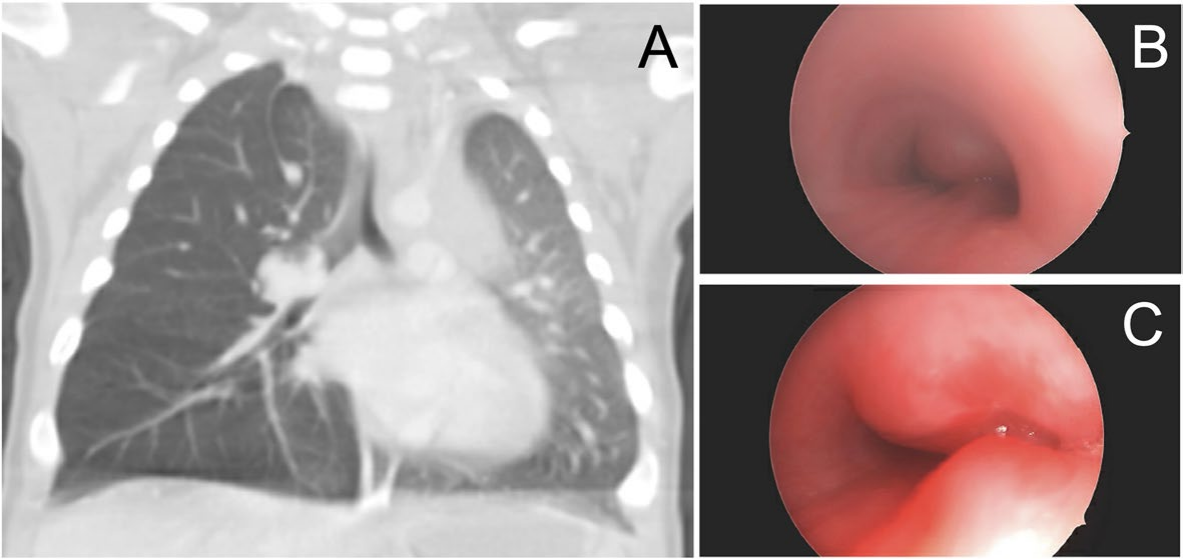
Lower airway infantile hemangioma as demonstrated by (**A**) chest CT showing mass compressing lumen of right mainstem bronchus, (**B**) bronchoscopy of proximal right mainstem bronchus on day of intubation demonstrating complete occlusion, and (**C**) bronchoscopy of proximal right mainstem bronchus after 6 days of treatment, demonstrating 70% occlusion

On the ward, he developed worsening work of breathing for which his high-flow nasal cannula was increased to 15 l per minute with 30% FiO2. Despite the increase in respiratory support, his work of breathing continued to worsen. A chest x-ray was obtained and demonstrated right lung hyperinflation suggestive of air-trapping, and he was transferred to the pediatric intensive care unit. Repeat imaging re-demonstrated hyperaeration of right lung, with leftward mediastinal shift (Fig. 1A). Due to concern for obstruction and inadequate ventilation, pediatric pulmonology was consulted, and a CT chest was performed. The CT scan demonstrated a right hilar vascular mass (Fig. 2A, 2x1.5 × 1.9 cm, 100% occlusion) compressing and occluding the right mainstem bronchus, hyper-expansion of the right lung, decreased left lung volume, and leftward mediastinal shift. Otolaryngology and pediatric surgery were consulted and given the location of the mass; recommendations were made to transfer patient to a subspecialty center for advanced airway management.

Upon arrival, the patient was found to be in severe respiratory distress progressing to acute hypoxemic and hypercarbic respiratory failure due to compression of the right mainstem bronchus and subsequent hyperinflation of the right lung. Initial intubation was complex, requiring placement of the endotracheal tube into the left mainstem bronchus following a single-lung ventilation strategy so as not to worsen the mediastinal shift. Extracorporeal membrane oxygenation (ECMO) was on standby, with a circuit outside the patient’s room, given the high risk of hypoxemia and cardiac arrest (Fig. 1B, Fig. 2B). Therapy for presumed infantile hemangioma was initiated with propranolol (initially 1 mg/kg/day divided three times daily (TID), increased over 3 days to maximum 3 mg/kg/day divided TID. Diagnosis was later confirmed with MRI. After initiation of beta blocker therapy, interval reduction in size of hemangioma was noted on flexible bronchoscopy (Fig. 2C). Over the next several days, oxygenation and ventilation improved with ventilatory support and aggressive airway clearance, and imaging demonstrated appropriate re-distribution of lung volumes. The patient was successfully extubated to simple nasal cannula without complication on hospital day 17 (Fig. 1C). The patient was discharged home breathing comfortably on room air and prescribed propranolol for maintenance therapy. Chest ultrasound prior to discharge noted that the vascular malformation had decreased in size (0.7 × 0.6 × 0.8 cm, ~ 70% occlusion). The patient followed up every 3 weeks with the multi-specialty vascular malformations clinic for serial imaging to monitor the size of the involuting infantile hemangioma.

## Discussion and conclusions

This is the first documented case of an infant with rapid deterioration and acute respiratory failure secondary to a lower, intra-airway hemangioma, who was managed safely with escalation to a tertiary care facility with appropriate subspecialists. This case provides a complete demonstration of initial general pediatrics presentation through ICU management and subsequent discharge. Solitary lower airway hemangiomas without skin findings are exceedingly rare but may present a diagnostic challenge for the general pediatrician given their symptom overlap with common diseases such as acute bronchiolitis or reactive airway disease. Given this child’s initial presentation with respiratory distress that was misdiagnosed as a viral respiratory infection, his clinical deterioration and subsequent clinical course offer multiple high yield learning points for general pediatricians. Intra-airway mass should warrant consideration among general pediatricians for infants with respiratory distress who do not progress as expected.

First, early escalation of care and transport to a setting with pediatric critical care and airway subspecialty services is critical. Due to the obstructive physiology of an intra-airway mass, including air entrapment from a ball-valve effect, delay in transport may lead to mediastinal shift, obstructive shock, and acute respiratory failure. Given these potential life-threatening complications, the transport of a patient with a suspected intra-airway mass should be supervised by a pediatric critical care transport team with expertise in advanced airway management. Similarly, referral to an ECMO center should be strongly considered.

Second, it is critical to assemble the appropriate personnel and equipment early, optimally within the first 24-48 hrs. Table 1 details the multiple teams involved upon the patient’s arrival to our unit and the approximate timing of their involvement. Given the patient’s risk of hypoxemic cardiac arrest, an ECMO circuit and team were standing by while pediatric airway specialists performed left mainstem intubation and subsequent bronchoscopy in the operating room. Pediatric pulmonology and anesthesia may be helpful to aid in single-lung ventilation strategies, both in the operating room and in the PICU. For this patient, our pediatric ECMO team remained on standby while the patient underwent lung recruitment, and beta blocker therapy. In the absence of a skilled pediatric airway specialist however, adjunct respiratory therapies may be considered to increase ventilation to the distal alveoli and bypass an obstructive airway mass, including heliox delivered through a high-flow nasal cannula device.

**Table 1:** Recommended team and subspecialist involvement

Service	**Initial stabilization (12-24 h)**	**Diagnostic workup and management (24-48 h)**	**Long-term management (≥48 h)**
Pediatric Critical Care Transport	**X**	**X**	
Pediatric Critical Care	**X**	**X**	
Pediatric Otolaryngology	**X**	**X**	
Pediatric Anesthesia	**X**	**X**	
Pediatric Surgery	**X**	**X**	
ECMO / perfusion	**X**	**X**	
Interventional radiology		**X**	
Pediatric Pulmonology		**X**	
Vascular malformation specialists			**X**
Dermatology			**X**
Hematology/oncology			**X**

Third, as definitive management depends on accurate diagnosis, it is imperative to involve pediatric radiology who may help advise appropriate radiographic studies to support the most likely diagnoses, as well as pediatric trained interventionalists (IR, Surgery, ENT) who can advise on appropriate strategies to obtain definitive tissue diagnoses. In the case of this patient, gross bronchoscopic examination was highly suspicious for intra-airway hemangioma, although MRI was necessary to confirm the diagnosis. It is important to consider that any transport for imaging or operative intervention in an infant with single lung ventilation incurs high risk of endotracheal tube dislodgement, inadequate ventilation, and acute cardiopulmonary arrest.

Following confirmation of congenital infantile hemangioma by MRI, beta blocker therapy was initiated with consensus of a multidisciplinary team. While beta blockade is standard of care for infantile hemangiomas, this treatment in infants may lead to circulatory compromise as infants rely on contractility to recruit cardiac output [12]. As blocking chronotropy may lead to hemodynamic instability, continuous cardiopulmonary monitoring and availability of appropriate inotropic support are necessary. For this case, propranolol therapy was initiated in the PICU where the patient was monitored for signs of end organ dysfunction in the context of sinus bradycardia.

Infantile hemangiomas are treatable causes of airway obstruction, but present challenging management strategies as infants may present with acute respiratory failure and incur significant bradycardia from therapy. With early referral to a tertiary care center and the recruitment of multiple pediatric subspecialty teams, infants who present in this rare manner may obtain timely diagnosis, life-saving treatment, and be restored to good health.

## Data Availability

N/A.

## Abbreviations

ECMO: extracorporeal membrane oxygenation
ENT: otolaryngology
heliox: helium-oxygen blended therapy
IH: infantile hemangioma
IR: interventional radiology
MRI: magnetic resonance imaging
PEEP: positive end expiratory pressure
PICU: pediatric intensive care unit.
